# Evaluating the Accuracy of Automatic Speech Recognition Systems in Home Healthcare Settings

**DOI:** 10.1093/geroni/igaf122.3438

**Published:** 2025-12-31

**Authors:** Dayoung Yu, Sasha Vergez, Grace Flaherty, Maryam Zolnoori, Nicole Onorato, Julia Hirschberg, Maxim Topaz, Margaret McDonald

**Affiliations:** VNS Health, New York, New York, United States; VNS Health, Bronx, New York, United States; VNS Health, New York, New York, United States; Columbia University, New York, New York, United States; VNS Health, New York, New York, United States; Columbia University, New York, New York, United States; Columbia University, New York, New York, United States; VNS Health, New York, New York, United States

## Abstract

Recent developments in automatic speech recognition (ASR) systems have resulted in increased use of automated transcription services to analyze verbal communications. We aimed to analyze the accuracy of AWS General Transcribe (AWS-GT) on audio recordings of home healthcare patients. These audio recordings included in-person clinician visits and phone calls with research assistants (RAs). Study staff rated the clarity of the audio using a scale of low, medium and high. The audio quality was similar among the two types of recordings. Our results are a part of a larger study aiming to use automated speech processing to identify risk factors for hospitalization and emergency department visits. Overall, 4002 utterances, defined as uninterrupted blocks of speech with four or more words, were analyzed— 3,472 (87%) from clinician recordings and 520 (13%) from RA recordings. Word error rate (WER; range 0(best)-1(worst)) was used to assess the performance of AWS-GT, compared to a gold-standard manual transcription. We found a statistically significant difference in WER for clinician recordings (.26) and RA recordings (.19) (p < 0.05). We also observed that the WER was poorest for short, four-to-eight-words, utterances (.39), and that this improved as utterances grew longer (p < 0.05). Clinician recordings (35%) had a greater number of short utterances than RA recordings (25%) (p < 0.05). This shows how differences in conversation dynamics can impact the accuracy of ASR systems. It is incredibly important that we determine whether existing software is accurate enough for use in clinical documentation and prediction models dependent on ASR systems.

